# Supplementary material for: The RNA Polymerase Inhibitor Corallopyronin A Has a Lower Frequency of Resistance Than Rifampicin in Staphylococcus aureus
Source: Antibiotics (Basel). 2022 Jul 8;11(7):920. doi: 10.3390/antibiotics11070920 (PMC9311656; doi:10.3390/antibiotics11070920)
Supplement: Supplementary file 1 [file antibiotics-11-00920-s001.zip › antibiotics-1792556-supplementary.pdf]

Supplemental Materials

The RNA polymerase inhibitor corallopyronin A has a lower frequency of resistance than rifampicin in *Staphylococcus aureus*

Jan Balansky[1,2], Kenneth Pfarr[1,2], Christiane Szekat[1,2], Stefan Kehraus[6], Tilman Aden[1,2], Miriam Grosse[3,4], Rolf Jansen[3,4], Thomas Hesterkamp[5], Andrea Schiefer[1,2], Gabriele M. König[6], Marc Stadler[3,4], Achim Hoerauf[1,2], Gabriele Bierbaum[1,2*]

Supplemental Table S1: Raw data set for the determination of mutation frequency and mutation rates shown in Table 2 in the manuscript using 1 ml of culture

Agar containing corallopyronin		Agar containing rifampicin	
CFU on CorA Agar/ml	CFU / ml Culture (no antibiotic)	CFU on Rif Agar/ml	CFU / ml Culture (no antibiotic)
4	2.65E+08	76	3.50E+08
4	2.75E+08	16	2.30E+08
0	3.10E+09	24	3.05E+08
8	1.90E+08	20	3.35E+08
0	2.80E+09	52	2.25E+08
0	1.85E+09	20	3.20E+08
4	2.05E+08	76	2.75E+08
4	2.25E+08	24	4.25E+08
4	2.90E+08	52	3.10E+08
7	3.15E+08	28	4.45E+08
3	2.65E+08	48	3.00E+08
8	2.35E+08	20	5.65E+08
2	2.75E+08	80	5.40E+08
4	2.65E+08	12	2.70E+08
5	2.60E+08	20	3.00E+08
7	2.40E+08	16	3.20E+08
5	5.75E+08	12	3.20E+08
6	1.90E+08	8	3.15E+08
14	3.70E+08	32	3.75E+08
2	2.75E+08		
3	1.70E+08		
8	3.25E+08		
1	3.95E+08		
4	4.40E+08		
1	1.10E+08		
7	5.15E+08		

Supplemental Table S2: Raw data set for the determination of mutation frequency and mutation rates shown in Table 2 in the manuscript using 0.1 ml of culture

Agar containing corallopyronin		Agar containing rifampicin	
CFU on CorA Agar/ml	CFU per ml Culture	CFU on Rif Agar/ml	CFU per ml Culture
70	2.65E+08	130	2.65E+08
20	2.75E+08	50	1.45E+08
30	3.10E+08	20	1.45E+08
10	1.85E+08	70	1.70E+08
20	2.05E+08	50	1.30E+08
40	2.25E+08	80	3.50E+08
10	2.90E+08	20	2.30E+08
20	3.15E+08	60	3.05E+08
10	2.75E+08	20	3.35E+08
10	2.65E+08	70	2.25E+08
10	2.60E+08	20	3.20E+08
10	1.90E+08	50	2.75E+08
10	3.70E+08	40	4.25E+08
10	2.75E+08	20	3.10E+08
10	3.95E+08	20	3.00E+08
10	4.40E+08	60	5.65E+08
0	2.40E+08	30	5.40E+08
0	3.70E+08	30	2.70E+08
0	4.80E+08	80	3.20E+08
0	4.20E+08	30	3.20E+08
0	3.20E+08	40	3.15E+08
0	1.60E+08	50	3.75E+08
0	2.60E+08	0	5.70E+08
0	3.40E+08		
0	2.20E+08		
0	2.70E+08		
0	3.00E+08		
0	2.70E+08		
0	9.00E+07		
0	2.30E+08		
0	2.70E+08		

Supplemental Figure S1: Decrease of the CorA isomer in agar plates during storage at different temperatures

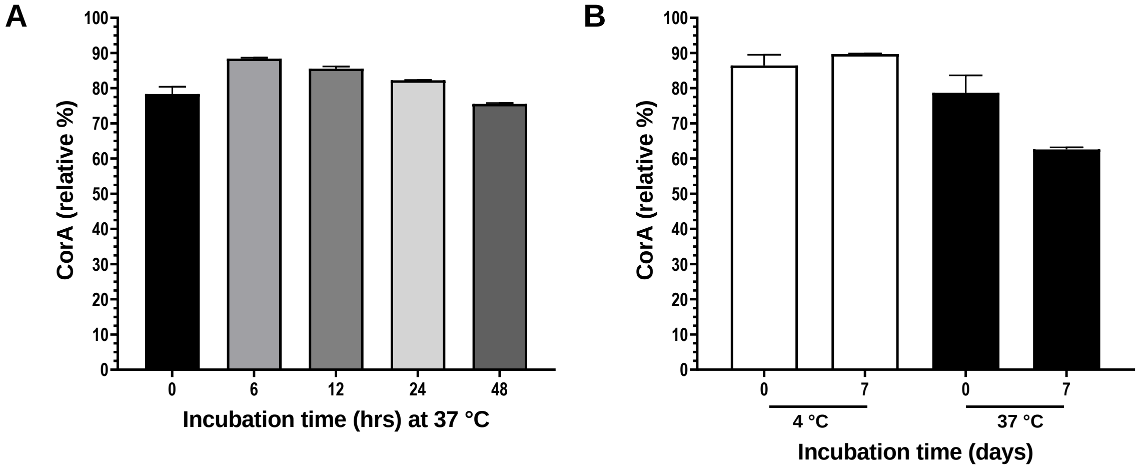

Supplemental Figure S1. CorA is stable in agar for 48 hours at 37 °C. A) Duplicate agar plates (8 mL) containing 1 mg/mL CorA were poured. After 0-, 6-, 12-, 24-, and 48-hours incubation at 37 °C, 1 cm^2 plugs were taken and frozen at -20 °C. B) After 0- and 7-days of incubation at 4 °C or 37 °C, agar plugs were taken and frozen at -20 °C. Agar plugs were extracted with 100% ethyl acetate by mixing at room temperature for 30 minutes. Ethyl acetate extracts were prepared for HPLC-DAD analysis as published (Krome et al., 2020). Relative levels of CorA to the CorA isomer CorA' were calculated as percent and means graphed. Figure generated with GraphPad Prism 9.4 for Windows, GraphPad Software, San Diego, California USA, www.graphpad.com.

Method: To test the stability of neat CorA in agar plates, GC agar plates (Becton-Dickinson, Heidelberg, Germany) containing 1 mg/L CorA were poured and stored at 37 °C for 24 and 48 h or at 4 ℃ and 37 °C for a week. The concentration of CorA was analyzed after extraction from the agar and mixing with acetonitrile. The extracts were analyzed by HPLC using an Alliance e2695 separation module and a 2998 PDA detector (Waters, Eschborn, Germany), employing a Waters XBridge® Shield RP18 column (3.5 µm, 2.1 × 100 mm, 130 A) and the following gradient: mobile phase: A (acetonitrile/water 5/95 with 5 mM ammonium acetate and 40 µL acetic acid per Liter); B (acetonitrile/water 95/5 with 5 mM ammonium acetate and 40 µL acetic acid per Liter); gradient from 70 % A / 30 % B to 20% A / 80 % B within 30 min; flow: 0.3 mL/min; column temperature: 30 °C. CorA was quantified via an external reference standard measured at 300 nm (Krome et al., 2020).

References

Krome, A., Becker, T., Kehraus, S., Schiefer, A., Steinebach, C., Aden, T., Frohberger, S.J., Marmol, A.L., Kapote, D., Jansen, R., et al. (2020). Solubility and stability enhanced oral formulations for the anti-infective Corallopyronin A. Pharmaceutics *12*, 1105. https://doi.org/10.3390/pharmaceutics12111105.
